# Advances and Controversies in Perioperative Airway Management

**DOI:** 10.1155/2016/1965623

**Published:** 2016-01-11

**Authors:** Pavel Michalek, Magboul M. Magboul, Kamil Toker, William Donaldson, Makoto Ozaki

**Affiliations:** ^1^Department of Anaesthesia and Intensive Medicine, 1st Medical Faculty, Charles University in Prague and General University Hospital, Ulice Nemocnice 2, 120 21 Prague, Czech Republic; ^2^Norwich Research Park, University of East Anglia, Norwich, Norfolk NR4 7TJ, UK; ^3^Department of Anesthesia, University of Iowa Hospitals and Clinics, Iowa City, IA 52242-1009, USA; ^4^Department of Anesthesiology and Reanimation, Medical Faculty, Kocaeli University, Umuttepe, 413 80 Kocaeli, Turkey; ^5^Department of Anaesthetics, Antrim Area Hospital, Bush Road, Antrim BT41 4RD, UK; ^6^Department of Anesthesiology, Tokyo Women's Medical University, Shinjuku-ku, Tokyo 162-8666, Japan

The appropriate, case- and patient-tailored selection of an airway management technique is one of the cornerstones of safe perioperative practice. Preparation for airway management begins with assessment of the airway and with the identification of any potentially challenging anatomical variations. However, the currently available clinical tests have quite poor prognostic value in the prediction of difficult intubation [1] and use of a combination of tests is recommended in order to gain better diagnostic value [2]. At present, airway evaluation and assessment fails to completely exclude the unexpected difficult airway in daily anesthetic practice. Recently, a strong focus has been placed on imaging methods: magnetic resonance imaging (MRI), airway ultrasound, and nasal endoscopy [3].

Various new airway devices have been introduced in order to improve perioperative airway management in the last decade. These devices vary from videolaryngoscopes to innovative supraglottic airway devices, fiber-optic scopes, optical stylets, optical tracheal tubes, airway exchange catheters, and bougies. The use of most of these novel devices in difficult airway management scenarios is not supported by any robust evidence, and they remain only as devices used mostly in teaching and mannequin settings and, apart from videolaryngoscopes, they are not included in any recent guidelines [4].

Difficult or failed intubations in perioperative airway management are relatively rare and occur much more frequently in prehospital medicine, Emergency Departments, or Intensive Care Units. However, critical situations such as “cannot intubate, cannot oxygenate” are often poorly managed and may be associated with significant morbidity and mortality [5].

The invention of novel supraglottic airway devices has been driven by efforts to supersede tracheal intubation, even for traditional indications such as in obese patients, advanced laparoscopic procedures, positions other than supine, or conditions associated with an increased risk of aspiration of gastric contents [6]. These devices should exhibit high oropharyngeal seal pressures and enable smooth insertion even in the hands of novice users. Another area of development of supraglottic airway devices concentrates on their use in difficult airway management.

This special issue of the journal focuses on progress, innovations, and controversial issues in perioperative airway management. The original investigations and review articles cover different angles of this extensive anesthetic subspecialty. One clinical study investigated the changes in the intracuff pressures in two different types of tracheal tubes depending on the head and neck positioning. This paper entitled “Comparison of Pressure Changes by Head and Neck Position between High-Volume Low-Pressure and Taper-Shaped Cuffs: A Randomized Controlled Trial” found that taper cuffs were associated with a lower rise in intracuff pressures than the other type during neck extension and flexion. This finding may be important for postoperative tracheal morbidity: increased intracuff pressures together with prolonged mechanical ventilation have been associated with an elevated incidence of tracheal ischemic lesions [7]. The clinical performance of five different supraglottic airway devices with increased aspiration protection (2nd generation) was investigated in the manuscript “Comparison of Five 2nd-Generation Supraglottic Airway Devices for Airway Management Performed by Novice Military Operators.” The authors concluded that the Laryngeal Mask Airway Supreme and i-gel performed better for most insertion parameters such as insertion time, ease of insertion, and success rate than the other three devices.

Simulation studies are an important part of anesthetic research; however extrapolating their findings into clinical medicine is problematic [8]. Mannequin airway studies have been repeatedly used as a first assessment tool for new devices and airway management techniques but their oropharyngeal anatomy may differ significantly from real patients [9]. Two studies in this special issue focus on simulation in airway management. Gum-elastic bougie improved the success rate of tracheal intubation in an infant model of difficult laryngoscopy when compared with a standard procedure performed with the Miller laryngoscope, in the article entitled “Utility of a Gum-Elastic Bougie for Difficult Airway Management in Infants: A Simulation-Based Crossover Analysis.” Two different types of videolaryngoscopes, McGrath and Pentax-AWS Airwayscope, and direct Macintosh laryngoscope were compared in simulated vomitus and hematemesis scenarios. This article “Comparison of Direct and Indirect Laryngoscopes in Vomitus and Hematemesis Settings: A Randomized Simulation Trial” favored Macintosh and McGrath laryngoscopes in terms of success rate and intubation time.

Bedside ultrasound has also been used within the last decade in perioperative airway management. In their review article, entitled “The Role of Airway and Endobronchial Ultrasound in Perioperative Medicine,” the authors recommend the use of ultrasound for location of the cricothyroid membrane, confirmation of tracheal tube placement, or location/biopsy of pulmonary nodules but do not suggest its routine use in preoperative prediction of the difficult airway. Some rare disorders may significantly affect airway management and perioperative ventilation. The paper entitled “Respiratory Strategies and Airway Management in Patients with Pulmonary Alveolar Proteinosis: A Review” summarizes the available data on lung isolation techniques for this procedure and recommends step-by-step ventilation strategies including extracorporeal oxygenation for these patients. Pediatric airway management may be associated with controversial issues such as prediction of difficult laryngoscopy, management of the difficult airway, selection of cuffed or uncuffed tracheal tubes, timing of extubation, and indications for the use of supraglottic airway devices. The review article entitled “Controversies in Pediatric Perioperative Airways” concludes that strong evidence regarding all these controversies is missing, most data has been extrapolated from adult airway management, and more randomized controlled trials on this topic are vitally needed. Supraglottic airway devices are alternatives to tracheal intubation in elective surgical procedures without additional risk of aspiration and may be also used as an alternative airway device in difficult intubation scenarios. The paper entitled “Complications Associated with the Use of Supraglottic Airway Devices in Perioperative Medicine” highlights the incidence and significance of complications related to these devices: aspiration, nerve injuries, trauma, and postoperative sore throat.

The field of airway management is still evolving. The incidence of life-threatening events and complicated scenarios is very low and these are not completely predictable. This fact explains the lack of robust clinical trials in airway management and it is also a reason why most guidelines and recommendations are based on expert opinions, consensus, and case series rather than on high-quality evidence-based medicine. International groups of experts such as the “Difficult Airway Research Collaboration” (http://www.darc-airway.com/site/) have been established in order to create and coordinate more robust pan-European and worldwide clinical trials in airway management. Future airway research should focus on setting up large multicentre studies which are adequately powered to evaluate the incidence of even very rare complications, such as aspiration of gastric contents or significant damage to anatomical structures caused by airway interventions.

*Pavel Michalek* *Magboul M. Magboul* *Kamil Toker* *William Donaldson* *Makoto Ozaki*

## References

[1] Lundstrom L. H., Vester-Andersen M., Moller A. M., Charuluxananan S., L'hermite J., Wetterslev J. (2011). Poor prognostic value of the modified Mallampati score: a meta-analysis involving 177, 088 patients. *British Journal of Anaesthesia*.

[2] Shiga T., Wajima Z., Inoue T., Sakamoto A. (2005). Predicting difficult intubation in apparently normal patients: a meta-analysis of bedside screening performance. *Anesthesiology*.

[3] Kuo G. P., Torok C. M., Aygun N., Zinreich S. J. (2011). Diagnostic imaging of the upper airway. *Proceedings of the American Thoracic Society*.

[4] Frerk C., Mitchell V. S., McNarry A. F., Mendonca C., Bhagrath R., Patel A. (2015). Difficult airway society 2015 guidelines for unanticipated difficult intubation in adults. *British Journal of Anaesthesia*.

[5] Cook T. M., Woodall N., Frerk C. (2011). Major complications of airway management in the UK: results of the Fourth National Audit Project of the Royal College of Anaesthetists and the Difficult Airway Society. Part 1: anaesthesia. *British Journal of Anaesthesia*.

[6] Michalek P., Miller D. M. (2014). Airway management evolution—in a search for ideal extraglottic airway device. *Prague Medical Report*.

[7] Touat L., Fournier C., Ramon P., Salleron J., Durocher A., Nseir S. (2013). Intubation-related tracheal ischemic lesions: incidence, risk factors and outcome. *Intensive Care Medicine*.

[8] Russo S. G., Bollinger M., Strack M., Crozier T. A., Bauer M., Heuer J. F. (2013). Transfer of airway skills from manikin training to patient: success of ventilation with facemask or LMA-Supreme by medical students. *Anaesthesia*.

[9] Schalk R., Eichler K., Bergold M. N., Weber C. F., Zacharowski K., Meininger D. (2015). A radiographic comparison of human airway anatomy and airway manikins—implications for manikin-based testing of artificial airways. *Resuscitation*.

